# The Body Speaks: Using the Mirror Game to Link Attachment and Non-verbal Behavior

**DOI:** 10.3389/fpsyg.2018.01560

**Published:** 2018-08-23

**Authors:** Rinat Feniger-Schaal, Yuval Hart, Nava Lotan, Nina Koren-Karie, Lior Noy

**Affiliations:** ^1^The Center for the Study of Child Development, University of Haifa, Haifa, Israel; ^2^The Graduate School of Creative Arts Therapies, University of Haifa, Haifa, Israel; ^3^School of Engineering and Applied Sciences, Harvard University, Cambridge, MA, United States; ^4^The Bob Shapell School of Social Work, Tel Aviv University, Tel Aviv, Israel; ^5^School of Social Work, University of Haifa, Haifa, Israel; ^6^Arison School of Business, Interdisciplinary Center (IDC), Herzliya, Israel

**Keywords:** mirror game, attachment, non-verbal behavior, exploration, dance/movement therapy, drama therapy

## Abstract

The Mirror Game (MG) is a common exercise in dance/movement therapy and drama therapy. It is used to promote participants’ ability to enter and remain in a state of togetherness. In spite of the wide use of the MG by practitioners, it is only recently that scientists begun to use the MG in research, examining its correlates, validity, and reliability. This study joins this effort by reporting on the identification of scale items to describe the non-verbal behavior expressed during the MG and its correlation to measures of attachment. Thus, we explored the application of the MG as a tool for assessing the embodiment of attachment in adulthood. Forty-eight participants (22 females, mean age = 33.2) played the MG with the same gender-matched expert players. All MG were videotaped. In addition, participants were evaluated on two central measurements of attachment in adulthood: The Adult Attachment Interview (AAI) and the Experience in Close Relationship questionnaire (ECR). To analyze the data, we developed the “MG scale” that coded the non-verbal behavior during the movement interaction, using 19 parameters. The sub-scales were reduced using factor analysis into two dimensions referred to as “together” and “free.” The *free* factor was significantly correlated to both measurements of attachment: Participants classified as having secure attachment on the AAI, received higher scores on the MG *free* factor than participants classified as insecure [*t*(46) = 7.858, *p* = 0.000]. Participants, who were high on the *avoidance* dimension on the ECR, were low on the MG *free* factor [*r*(48) = −0.285, *p* = 0.007]. This is the first study to examine the MG as it is used by practitioners and its correlation to highly standardized measures. This exploratory study may be considered as part of the first steps of exploring the MG as a standardized assessment tool. The advantages of the MG as a simple, non-verbal movement interaction demonstrate some of the strengths of dance/movement and drama therapy practice.

## Introduction

From the day a baby is born, the experience of relating to others is present and the complex weave of self in relation to others is being built. The significant interaction between the caregiver and the infant consolidate the “schema of being with" (Stern, 1983) long before language is available. The earliest learning about relationship, hence, is implicit, through the body, involving non-verbal behavior (Payne, 2017).

While the systematic assessments of relationships in childhood relay heavily on observation including verbal and non-verbal account (i.e., the strange situation procedure; Ainsworth et al., 1978; the emotional availability scales; Biringen et al., 2000), to date, the systematic examination of relationships in adulthood presented in the literature is based mostly on verbal report as the main source of information. In this preliminary study, we look at non-verbal interaction in adulthood using a common imitation exercise, the mirror game (MG), used in drama therapy and dance/movement therapy. We hypothesized that dyadic interaction in the course of play and movement holds valuable information that is linked to attachment style. In the present study, we used the MG to examine the non-verbal expressions of attachment, connecting between a solid psychological construct to the practice of drama therapy and dance/movement therapy.

### Drama Therapy and Dance/Movement Therapy

One of the contributions of Creative Arts Therapies )CATs) to psychotherapy is the emphasis, in addition to explicit verbal communication, on other means of expressions such as the use of images, metaphors, sounds, choices of materials, rhythms, movement, playfulness, etc. (Cattanach, 1999). These means of communication and assessment provide opportunities to work with populations that are limited in their verbal expression (such as people with Intellectual Disabilities) and to deepen the understanding with all client groups, regarding implicit processes and information.

The body as a means of communication is not a new notion. In recent years, however, the body and its relation to emotions (Fuchs and Koch, 2014), cognition (Gallese, 2005; Ziemke, 2016), interpersonal relationship (Vicaria and Dickens, 2016), and therapeutic processes (Ramseyer and Tschacher, 2011; Koole and Tschacher, 2016) have received greater attention both in the clinical practice and in research (Payne, 2017). There is a growing emphasis on the notion that body movement, and non-verbal behavior are another important channel to investigate, not only with children, or non-verbal populations, but as an important means by which we can gain insight into the way human beings interpret, express, and interact with the world around them.

The use of the body as a central component in the therapeutic processes is inherent to both drama therapy and dance/movement therapy. *Drama therapy* is an active, experiential approach that facilitates change through the core elements of drama and theater, i.e., play, role, narrative, and performance (Jennings, 1992; Jones, 2007). *Dance/movement therapy* is the psychotherapeutic use of movement and dance to promote emotional, social, cognitive, and physical wellbeing (American Dance Therapy Association [ADTA], 2016). Hence, for drama therapists and dance/movement therapists the non-verbal behavior is an integral part of their practice, using various techniques to elicit this kind of expression.

### The Mirror Game

Imitating, mirroring, or “joining” the other person’s movements or gestures are examples of common techniques used in dance/movement therapy (Koch et al., 2015; Koehne et al., 2016) and in drama therapy (Johnson, 2009). The MG used in this study is an exercise in imitation that has a clear structure. Players imitate each other’s movements in three rounds, which make it possible to experience different roles and interactions: in the first round one player leads and the other follows, in the second round they switch roles, and in the last round there is no designated leader or follower. The MG is commonly practiced in theater, drama therapy (e.g., Boal, 2013), and dance/movement therapy (McGarry and Russo, 2011), and it is used to enhance empathy and emotional understanding of others, and to promote participants’ ability to enter and remain in a state of togetherness (Schechner, 1994).

Although the MG is common practice in drama and dance/movement therapy, only recently has it become the target of thorough scientific scrutiny, reflecting the gap between clinical practice and empirical evidence. Interestingly, most studies on the MG were conducted by researchers from various disciplines other than arts therapies (i.e., physicists, computer scientists, neuroscientists), who showed a growing interest in using the MG as an experimental paradigm for measuring states of “togetherness” (Noy, 2014).

Some early studies on the MG used a device whereby players move handles along parallel tracks in one dimension, which provides automated quantitative indicators for the quality of interaction during the MG (Noy et al., 2011; Hart et al., 2014). The first of these studies found that players showed intervals of “togetherness motion” in which motion was complex, smooth, and synchronized. Togetherness motion occurred most frequently when no leader or follower was designated (Noy et al., 2011). This work on the MG received a comment in *Nature* (Shadan, 2011) owing to its pioneering contribution, which made possible the quantification of the encounter between two players.

Subsequent works found correlations between physiological parameters and the experience of togetherness in the MG (Noy et al., 2015b); studied the individual vs. shared characteristics of motion (Hart et al., 2014; Noy et al., 2015a); developed a computerized version of the MG with implications for rehabilitation (Zhai et al., 2014); used the MG to measure the link between synchrony and improvisation (Gueugnon et al., 2016); used the MG as a socio-motor biomarker for schizophrenia (Słowiński et al., 2017); and explored group dynamics during the MG (Himberg et al., 2018).

What are we missing by not having the CAT perspective in MG studies? All dyadic studies on the MG mentioned above used a machine or computerized version of the MG. The rich, clinical version of the MG, as commonly used by dance/movement therapists and drama therapists, has not been studied systematically to date. Following the methodology of the exact sciences, previous MG studies used a reduction of the data of the movement interaction, which enable an accurate quantification of the motion encounter. The present study sought to examine the MG in the way it is used in clinical settings (hence, full body mirroring, with no machine involved) and to validate its richness and complexity. To do so, we relied on knowledge of dance movement and drama therapy in the data analysis of the full-body MG.

### The MG and Attachment

Because the MG is first and foremost an interpersonal exercise, we sought to map the expressions of the interpersonal encounter during the MG, and to connect the MG to one of the most influential theories on human relationship: attachment theory (Bowlby, 1969/1982; Ainsworth et al., 1978). According to attachment theory, human beings are equipped with an attachment behavioral system that evolved to ensure proximity to a caregiver who provides (especially to young children) protection and assistance in times of distress (Shaver et al., 2000), and a “secure base from which to explore the world” (Ainsworth, 1964, p. 54). Attachment behavior conveys a social system in which confidence in the availability and responsiveness of close others organize contact-seeking and exploratory behavior (Crowell et al., 2002).

A central tenet of attachment theory is that individuals differ in their quality of attachment varying in secure vs. insecure attachment. Differences formed in the course of early child–caregiver relationship facilitate the development of mental representations of self and other, known as the internal working model (IWM, Bowlby, 1979). These IWM organize feelings, thoughts, and behavior over the life span (Bowlby, 1979; Miljkovitch et al., 2015).

Consolidation of the IWM and of the attachment patterns begins with the first relationship between child and caregiver. This early relationship is built on a series of body-to-body interactions. The somatic experience is the primary source from which children gain knowledge about emotions and relationships with others (Damasio, 1994). The ways in which the caregiver interacts and attends to the needs of the child are the main source for the implicit knowledge regarding the self in relation to others. Thus, the sense of self in relation to the other is first and foremost a body sense (Ogden and Fisher, 2015).

While the majority of approaches to the assessment of attachment in childhood depended heavily on observation of behavior that takes into account non-verbal information, studies of attachment in adulthood focused mainly on verbal account, interviews, and selfreport (Crowell et al., 1999; Farnfield and Holmes, 2014). In the present study, we used play for assessment of adults’ participants and observe the behavior in the game. The CATs have adopted an approach suggesting that movement, play, and the use of imagination are not attributes that belong exclusively to childhood. The use of movements and games as part of assessment and intervention, connecting body, movement, and attachment is therefore inherent in drama therapy and dance/movement therapy.

The topics of attachment and its non-verbal expressions in adulthood have received little research attention. Some examples include studies that investigated the correlation between personal space and attachment classification (Kaitz et al., 2004). Studies exploring attachment classification and body response to lexical stimuli (Fraley and Marks, 2011) found that attachment classification is correlated to non-verbal expression like comfort with distance, and the action of pushing and pulling. Some of the clinical literature has also described expressions of attachment in body and movement (e.g., Schore, 2011; Porges, 2011; Damasio and Carvalho, 2013; Ogden and Fisher, 2015). Hence, there is a lack of experimental paradigms for studying attachment in adults based on non-verbal expressions

In our first work on the topic of the MG and attachment, we found a correlation between the way people played the one-dimensional MG, using the MG device, and their attachment classification (Feniger-Schaal et al., 2015). Based on the kinematic measures indicated by the device, we found that people with secure attachment played a more complex and less synchronized game, than did people with insecure attachment. These results reinforce the notion of exploration and openness as an important attributes of attachment security. Our results showed that the two behavioral systems of attachment and exploration intertwined (Bowlby, 1979, 1969/1982; Ainsworth et al., 1978; Elliot and Reis, 2003).

The MG device enabled high-resolution measures that showed significant results that connected attachment classification with microanalysis of movements. However, reduction of the interaction using the MG device is limited in its clinical implications. Furthermore, the measurement device allows limited expression of the IWM because it is constrained to movement in only one dimension. Therefore, we sought a lifelike, naturalistic, and rich interaction, applicable to clinical practice, so that therapists using the MG would have a systematic way of gaining information based on the MG, and of planning their interventions with the MG. From a research perspective, the simplicity of full-body MG, which requires no special technology or equipment, has potential value for future investigations.

### The Present Study

The aim of the present study was twofold: to develop a way of analyzing the non-verbal interaction during the MG and to validate it using key measurements of attachment in adulthood: the Adult Attachment Interview (AAI; George et al., 1985/1996, unpublished reference) and the Experience in Close Relationship questionnaire (ECR; Brennan et al., 1998).

We hypothesized that the MG includes elements that provide access to the non-verbal expression of attachment patterns. There are at least two such elements. First, because the MG involves interpersonal encounter, it can activate the participant’s procedural knowledge about how to interact with another person (regulate emotions, search for proximity, or synchronize with the other). Second, the MG entails exploratory behavior in which participants play together and search for various patterns of movement. The MG can therefore tap into the participant’s IWM, especially in the first stage of the game, when the participant leads and must both initiate motion and to make sure not to “lose” the follower. In this role, the participant must negotiate needs second by second, which presents an opportunity to assess attachment-related behavior. In this pilot study, we explored the ways in which different attachment styles are manifested in the non-verbal encounter of the MG.

## Materials and Methods

### Ethics Statement

The Institutional Review Board (IRB) at the University of Haifa approved the described experiments, including the written consent procedure (approval number 086/13). All the participants provided written informed consent to participate in the study.

### Participants

Forty-nine participants started the study. One participant quit at the interview session, therefore we analyzed the data for 48 participants, 22 females, mean age = 33.2 (*SD* = 7.3), mean number of years of education = 19.5 (*SD* = 2.6). All participants were Israeli Jews. 44(91.7%) spoke Hebrew as their first language, four others spoke either English, Russian or other European language. Four participants (8.3%) defined themselves as Orthodox Jews, Five others (10.4%) defined themselves as Conservative Jews, 39 (79.59%) defined themselves as non-religious. Twenty-seven of the participants were married, nine were in a relationship, four were divorced, and eight were single. Thirty-eight had no previous experience with the MG, nine had some, and one participant had extensive experience with the MG. Thirty-six had no improvisational experience of any kind (movement, music, drama), 10 had some experience in improvisation, and two had extended experience (see the descriptive statistics of the participants in **Supplementary Table S1**). Participants were students and staff at the Weizmann Institute of Science, who volunteered to take part in the study.

### Procedure and Measures

Participants attended two sessions. In the first session, they played the MG with a gender-matched expert player (research assistant) aiming to control for gender differences in movement (Bente et al., 1998; Lozza et al., 2018). In the second session, participants completed the ECR questionnaire and were individually administered the AAI. This study was part of a larger project that explored the MG paradigm to study adult interaction (see Hart et al., 2014; Feniger-Schaal et al., 2015, Feniger-Schaal and Lotan, 2017).

#### The Mirror Game

**FIGURE 1:**
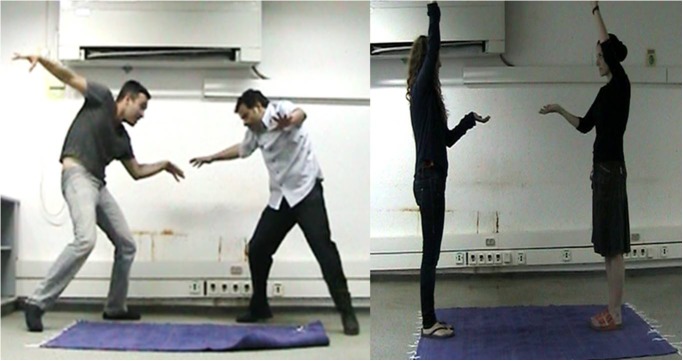
Examples of the MG: each participant played with a research assistant of the same gender. Note: all participants shown in this figure provided written informed consent for the publication of this image.

Participants were instructed to play the MG, which involved mirroring each other’s movements while assuming the different roles of leader and follower (see **Supplementary Material** for the complete MG instructions, and **Figure 1** for examples of the MG). The MG consisted of three rounds of five minutes each: in the first round the participant led, in the second the experimenter, and in the last round there was no designated leader. All games were videotaped. In this study we focused mainly on analyzing the first round, in which the participant led and the research assistant was following. This round is the first interaction between the participant and the experimenter, and therefore movement is less biased by leadership on the part of the expert player. In addition, one measure in the MG scales reflected the complete game and assessed whether there were any differences between the first round and the other two rounds. We also evaluated the third round and examined the shifts between the role of leader and follower.

#### The MG Scales

The MG scales (MGS) were developed for the purpose of the present study. The question that guided our observation of the games was: How is it for the participant to meet with another person? In the first stage, two researchers watched the videos and identified various components that pointed to individual differences in the MG. This process involved bottom-up (based on the video) and top-down (based on theory) method.

The identified components were then defined as a scale ranging 1–5 (1 = the negative end of the scale, 5 = the positive end). The development of the scales was based on theories used in dance/movement practice and on the observation method derived from attachment studies. More specifically, the definitions of the scales were influenced by the Emotional Availability Scales, which are commonly used to analyze parent–child interaction in attachment-related studies (EAS; Biringen et al., 2000), the Strange Situation Procedure (Ainsworth et al., 1978), Laban Movement Analysis (Laban, 1975; Levy and Duke, 2003), and Kestenberg Movement Profile (Kestenberg-Amighi et al., 1999/2018). We also resorted to our clinical experience as drama therapists and dance/movement therapists. In general, we were searching for communication of affect and for various ways of expressions relating to the encounter with the partner in the game.

Next, three “new” coders (hence, not the researchers that initially developed the scales) watched the MG videos and strived to reach reliability. Only the scales that – reached reliability were included in the final coding system. This process resulted in 19 scales that are described in the *MG Scales* manual (Feniger-Schaal et al., 2015, unpublished reference) and summarized in **Table 1**. **Supplementary Table S2** presents the inter ratters reliability if the MG behavior scales. The coding system focused on the following themes: *body movements*: which body parts participate in the game, which planes of movement were used and explored, the use of personal distance; *quality of movement*: tension, flow, pace; *exploration:* how rich and versatile the movements were*; affect*: negative affect, having fun, sharing affect; *minding the other*: making reference to the other, connecting and disconnecting eye contact, gaze aversion, arching, competing with or teasing the other; *unusual behavior*: behavior that seems odd or bizarre in the context of the MG.

**Table 1:** The MG scales.

The Scale	Description	Scoring
1. The “greeting”	Coding the first 45 s of the game, the way the player presents him/herself and begin the game	High score for appropriate checking of the encounter with the other and adapting to the beginning of the game
2. Breaks	Coding the times when the MG stops (breaks), i.e., the participant asks questions (after the first minute), burst into laughter or tears, or stops the game in any other way	High scores for no breaking of the game
3. Flow/shift	The flow of the movement	High score for flow of movements that seems to emerge from the previous movements
4. Pace	The pace of movement (changes from slow to fast)	High score for a pace that the partner can follow
5. Body parts	The use of the different parts of the body: limbs vs. the center of the body; robotic movements vs. soft and round movements in which the joints are used	High score for rich use of the body including the center of the body, and the performance of shape like movements (as opposed to robotic)
6. Directions of movement	The use of different movement planes: vertical, sagittal, and horizontal.	High score for the use of combinations of planes
7. Distance	The distance between the players	High score for exploring different distances between the players
8. Tension/relaxed	Physical indication of tension in the body and face, for example flexing the shoulders, or furrowing the eyebrows	High score for relaxed, no-tension affect
9. Negative affect	Facial expressions of negative affect such as anger, boredom, irritation	High score for mostly positive affect
10. Having fun	Enjoying the encounter, positive affect, playfulness, having fun playing together	High score for the player appearing to enjoy most of the game
11. Shared affect	The players sharing affect like a smile or a facial expression that expresses moments of shared positive emotion	High score for moments of sharing positive affect
12. Competitiveness/teasing	Movement that calls for competition and even a sense of teasing	High score for little competitiveness or teasing
13. Reference to the other	Looking at the other to see whether the partner can follow the movement	High scores for referring to the partner during the game and checking the partner’s ability to follow
14. Arching	Stretching the back backward in a way that disconnects eye contact	High score for no arching
15. Eye contact	Making eye contact	High score for eye contact during most of the game
16. Gaze aversion	Using movements (other than arching) that actively disconnect eye contact	High score for no gaze aversion
17. Exploration	The extent to which the participant explores a variety of movements	High score for exploratory game in different dimensions (pace, space, use of the body, etc.)
18. Unusual behavior	Performing unusual behaviors during the MG, for example, pretending to sleep throughout the game or moving only the pelvis for the entire game	Dichotomous scoring for the presence or absence of unusual behavior
19. Leader/follower	Coding the roles the participant assumes in the third round of the game: whether the player takes the lead, follows, or there is a constant shifting between roles	High score for balanced shifting between the roles of follower and leader

#### The Adult Attachment Interview

This hour-long semi-structured interview involves a series of questions about childhood relationships with one’s parent’s respondents support their descriptions of relationships with specific episodic memories (George et al., 1985/1996, unpublished reference). Respondents are also asked about possible bereavement and abuse. The interview is transcribed verbatim and coded using the Main and Goldwyn (1998, unpublished reference) system. Individuals are assigned to one of three main classifications, based on their discourse during the AAI. The secure-autonomous (F) classification is associated with responses that are coherent, clear, relevant, and reasonably succinct. Participants labeled *F* are generally “free to explore” their childhood memories, both good and bad. The main characteristics of the Secure-Autonomous group are an open and flexible manner of exploring their childhood experiences, and the way these experiences influenced them as they are today. The transcripts of Insecure-Dismissing individuals (*DS*) tend to be characterized by idealization (overly positive generalizations not substantiated by specific memories) and/or insisting on their inability to recall specific memories. Dismissing individuals also tend to rely mostly on themselves and to minimize the significance of past experiences. This group is characterized by rigidity and lack of openness to explore various points of view regarding their childhood experiences. The narratives of insecure-preoccupied (*E*) individuals are typically lengthy, emotionally charged, and lack relevance and coherence. They may also display a passive tone and could be difficult to follow. Additional classification is the Unresolved-Disoriented (U), which shows signs of disorientation when discussing potentially traumatic events. Unresolved transcripts were also assigned a secondary classification (autonomous, dismissing, or preoccupied), which best describes the discourse when not discussing loss or abuse. Finally, the *cannot-classify* (CC) classification indicates a text narrative that does not fit into any organized (DS, E, or F) AAI placement. This is most often the case when the text demonstrates a striking or unusual mixture of mental states (Hesse, 1996).

The interviews were transcribed verbatim, and identifying information was removed before coding. Transcripts were coded by Nina Koren-Karie, a certified AAI coder trained by Mary Main and Erik Hesse. For reliability, a second certified AAI coder scored 21% of the interviews. Both coders were blind to all other project data and to the analysis and scores of the other coder. The rate of agreement across the five classifications, based on 21% of transcripts, was 97%, κ = 0.96, *p* < 0.01. Intraclass correlation (ICC) between the two coders’ scores was 0.88.

#### Attachment Orientations

Attachment orientations were assessed with a Hebrew version of the Experiences in Close Relationships questionnaire (ECR; Brennan et al., 1998). Participants rated the extent to which each item was descriptive of their feelings in close relationships on a 7-point scale, ranging from 1 = Not at all to 7 = Very much. Eighteen items assessed attachment anxiety (e.g., “I worry about being abandoned”), and 18 assessed avoidance (e.g., “I prefer not to show a partner how I feel deep down”). The reliability and validity of the scales have been repeatedly demonstrated (Brennan et al., 1998; for a review, see Mikulincer and Shaver, 2005). In our study, Cronbach’s alphas were high (0.84 for anxiety and 0.85 for avoidance). Mean scores were computed for each scale, and the two scores were not significantly correlated (*r* = 0.093, *p* = 0.53).

## Results

### AAI Analysis

All participants were interviewed, and the interviews were scored using the standard methodology. Participants were classified as secure (*F*, 22 participants, 45.8%) or insecure. In the insecure category, most were in the subcategory of insecure-dismissive (DS, 21 out of 26). Three participants had an insecure-preoccupied (E) classification, one an unresolved classification (U), and one interview was assigned to the “cannot classify” (CC) group. Because of the small number of participants in the E, U, and CC groups, we opted for comparing between the secure and insecure groups.

### Attachment Orientation

Based on the ECR questionnaire, we found that the mean score of avoidance in our sample, on a scale of 1–7, was 2.65 (*SD* = 0.868), and the mean score for anxiety was 3.33 (*SD* = 1).

### Attachment Scores and Background Variables

First, we tested for correlation between background variables and attachment measures. We found no significant correlation between the attachment scores and demographic variables (language, family status, religious beliefs, socioeconomic status, previous experience with improvisation, MG, drama, or dance movement, exercise routine, number of years of education, marital status, mother tongue, and number of children). The only exception was a significant gender difference in the ECR questionnaire for avoidance scores [*t*(31.85) = 2.2, *p* = 0.035, *d* = 0.73], but not for anxiety [*t*(32) = 0.53, *p* = 0.59]. The mean avoidance score for men (*M* = 2.94, *SD* = 1.02) was higher than for women (*M* = 2.33, *SD* = 0.58.), as has been found in other studies (see meta-analysis regarding ECR and gender differences, Del Giudice, 2011). Therefore, we entered gender as a covariate in the following analysis.

#### Links Between AAI and ECR

No significant differences were found between the AAI secure vs. insecure classification on the anxiety [*t*(46) = 1.5, *p* = 0.25] and avoidance [*t*(46) = −1.6, *p* = 0.12] dimensions.

### MG Analysis

**Table 2** presents the descriptive statistics of the MG scales.

**Table 2:** MG scales mean and SD.

Mirror Game Scale	Mean	*SD*
Greeting	4.52	0.87
Breaks	3.5	1.53
Fluent/shifts	3.81	1.28
Pace of movement	4.17	1.14
Body parts	4.04	1.32
Directions of movement	4.23	1.23
Distance	4.22	1.24
Tension/relaxation	4	1.30
Negative affect	3.85	1.2
Having fun	3.23	1.29
Shared affect	3.5	1.68
Competitiveness/teasing	4.56	0.99
Reference to the other	3.94	1.31
Arching	4.08	1.54
Eye contact	3.73	1.14
Gaze aversion	3.21	1.46
Exploration	2.92	1.35
Leader/follower	3.81	1.54

*Unusual behavior* was the only category measured on a dichotomous scale because we could not define a range. Either the behavior looked exceptionally bizarre or not. Eight participants (16.6%) showed unusual behavior during the MG.

**FIGURE 2:**
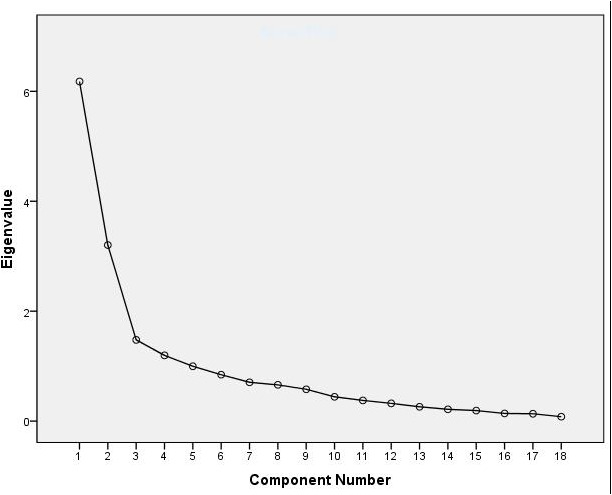
Scree plot showing the number of components that explain the variance in MG behavior. A factor analysis was conducted on 18 scales of MG behavior. The scree plot shows that two of these factors explain most of the variability, as the line begins to straighten after the second factor.

Next, we performed factor analysis to identify subgroups of variables that tend to vary together, and in this way reduce the scales into a few main domains [we used also a robust principal component (PC) analysis that yielded the same results]. We did not include in this analysis the *unusual behavior* scale because of its binary character. We used a varimax rotation for the procedure, which assumes that non-zero correlations between the factors are theoretically tenable and plausible. We found that the first two factors explain 52% of the variance (PC1: 34%, and PC2: 18%, see **Figure 2**).

The first PC, which we named *free*, included MG scales that reflect how easy or difficult it is for the player to play, and how open the player is to the experience of the game. The scales included in this subgroup relate to affect (for example: having fun, negative affect, tension*)*; and scales that capture how explorative the player is (for example, the use *of different movement directions and distance*, *different body parts*, *exploration* of different movement patterns, etc.). The second PC, which we named *together*, included MG scales that focus on the way people were engaged in the encounter with the other (for example, eye contact, gaze aversion, arching, greeting, making reference to the other, teasing or competing with the other, and shifting between the roles of follower and leader) (see **Table 3** for the loading of the different scales on each of the PCs).

**Table 3:** Loading of the MG scales on the two (PCs): free and together.

	Component	
	Free	Together
Greeting	0.188	**0.782**
Breaks	−0.076	**0.565**
Flow/shift	0.340	**0.554**
Pace of movement	0.128	**0.501**
Tension	**0.765**	−0.027
Distance	**0.551**	0.463
Eye contact	0.194	**0.728**
Gaze aversion	0.174	**0.737**
Arching	0.138	**0.519**
Having fun	**0.826**	0.326
Negative affect	**0.795**	0.277
Shared affect	**0.752**	0.312
Body parts	**0.773**	−0.134
Movement direction	**0.755**	−0.105
Reference to the other	0.258	**0.687**
Exploration	**0.635**	0.197
Competitiveness	−0.330	**0.485**
Leader/follower	−0.192	**0.685**

*^∗^Rotation Method: Varimax with Kaiser normalization. ^∗^The higher loading of the two PCs marked in bold.*

### Attachment Classification and MG Behavior

**FIGURE 3:**
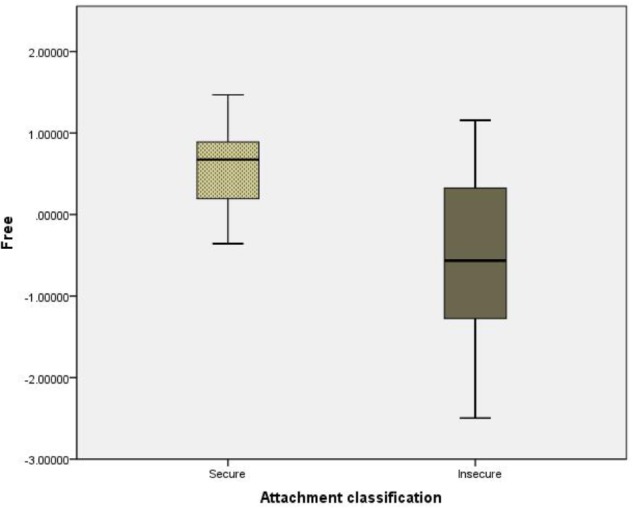
Difference between secure and insecure participans on the free MG behavior. Distribution of the *free* MG behavior among participants with secure and insecure attachment styles. Box-Whisker plots of the distributions of the free measure for secure (*N* = 22) and insecure (*N* = 26) participants are shown. Participants were tagged as secure/insecure based on their score on the AAI. The dotted gray (for secure) and dark gray (for insecure) boxes span the range from the first to the third quintiles, around median values (black line). The whiskers represent the minimal and maximal values.

Because some of the measures were not normally distributed, we used Mann–Whitney tests in the analysis to calculate the difference between secure and insecure attachment on the two MG behavior factors. Results show a significant difference between the secure and insecure groups on the *free* MG behavior (*U* = 116, *n*1 = 22, *n*2 = 26, *p* = 0.000, *r* = −0.16), but not on the *together* (**Figure 3** illustrates the distribution of the MG behavior *free* for participants with secure vs. insecure attachment classification). Participants classified as secure on the AAI, received higher scores on the MG behavior *free* factor than did participants classified as insecure.

We also examined the differences in the dichotomous *unusual behavior* measure and its connection to attachment classification. Using a chi-square test, we found a significant difference, so that all the eight participants who performed *unusual behavior* during the MG receiving an insecure classification on the AAI [*x*^2^(1) = 8.123, *p* = 0.004].

Next, we examined the correlation between the ECR dimensions, *avoidance* (controlling for gender) and *anxiety*, and the two MG behavior factors, *free* and *together*. Results show significant correlation between the avoidant dimension and the free factor (*r* = –0.285, *N* = 48, *p* = 0.007), so that participants who were high on the *avoidance* dimension were low on the *free* factor. Bootstrap results showed a 95% confidence interval lower limit of –0.637, and upper limit of –0.148. For the *anxiety* dimension, the results were non-significant (*r* = 0.271, *N* = 48, *p* = 0.06), but only a trend toward significance showing that participants who were high on the anxiety dimension were also high on the *free* factor. Neither the *anxiety* nor the *avoidance* dimension correlated significantly with the *together* factor. Comparing *unusual behavior* with results for the *anxiety* and *avoidance* dimensions showed that participants who performed unusual behavior were significantly lower on the *anxiety* dimension (*U* = 85, *n*1 = 8, *n*2 = 40, *p* = 0.03, *r* = −0.3), but showed no significant difference on the *avoidance* dimension.

Finally, we used regression to examine whether two different attachment measures, AAI and ECR, contribute to predict the MG behavior beyond the contribution of gender. As shown in **Table 4**, a significant regression equation was found [*F*(3,44) = 11.67], *p* < 0.001 with an overall effect size *R^2^* = 443. The AAI classification (secure or insecure) and the ECR avoidant dimensions (low or high on avoidance) each contribute independently to explain the variation in MG behavior on the *free* factor.

**Table 4:** Multiple regressions predicting free MG behavior.

		*B*	*SE B*	*β*
Gender		−0.589	0.236	−0.297^∗∗^
	AAI classification	−0.903	0.230	−0.455^∗∗∗^
	Avoidance	−0.434	0.141	−0.377^∗∗^
*R*^2^			0.443	
*F*			11.673^∗∗∗^	

*^∗^p < 0.05; ^∗∗^p < 0.005; ^∗∗∗^p < 0.001.*

## Discussion

Studies of attachment in adulthood tend to rely heavily on verbal reports. Non-verbal expression, however, may carry valuable information on interpersonal interactions, and therefore it serves as yet another channel for exploring expressions of attachment. This study used play and movement of adults to examine correlates of attachment. The results point to a connection between the MG behavior and two central measures of attachment, the AAI (George et al., 1985/1996, unpublished reference), and the ECR questionnaire (Brennan et al., 1998). Thus, movement interaction during dyadic play revealed information that is linked to attachment style.

Attachment is first consolidated during non-verbal interaction in childhood. The social engagement system is built upon a series of face-to-face, body-to-body interactions with an attachment figure. This implicit relational knowledge begins to be represented long before the availability of language and continues to operate implicitly throughout life (Lyons-Ruth et al., 1998). It is therefore no surprise that during an encounter people express their attachment style also non-verbally. Recognition of non-symbolically based representational system expressed through behavior has been a central contribution of infant research (e.g., Ainsworth et al., 1978; Tronick, 1989; Beebe and Lachmann, 1994). The present study characterizes non-verbal expressions in a dyadic encounter in adulthood by providing preliminary evidence of the connection between non-verbal behavior and attachment classification.

The findings of the present study show that the main component differentiating between secure (on the AAI) and low avoidance (on the ECR) participants on one hand, and insecure and high avoidance participants on the other, is the capacity to play in a “free” way, hence, playing in a flexible and explorative way with positive affect. Participants with secure attachment showed higher scores on the scales of having fun, rich use of body parts, and movement planes, displayed more shared affect with the other, and demonstrated a more exploratory game than did participants with insecure attachment. Furthermore, secure, low-avoidant participants showed lower negative affect and tension than did insecure participants. Using statistical methods, we grouped these non-verbal expressions and called it *free* to explore. Mary Main (George et al., 1985/1996, unpublished reference) marked the secure-autonomous group on the AAI with the letter *F* to indicate that the main characteristic of this group is being “free to explore” their childhood memories and their mental world. In the present study, we expanded this understanding into the physical world, showing that people with secure-autonomous attachment classification are available to use their body in a more flexible, complex, relaxed, and open way during an interpersonal encounter. Hence, the sense of freedom, with a positive affect, and the ability to be physically explorative was found to be a central feature of the secure group.

Exploration emerged as a significant component that correlates with security of attachment. This is not surprising, as the exploration behavior system is deeply rooted in Bowlby’s theory (Bowlby, 1969/1982). A central tenet of attachment theory is that the operation of the attachment system is closely intertwined with that of the exploration system (Ainsworth et al., 1978), and the link between attachment classification and exploratory play found in our results supports this idea. Other research has found empirical support for the centrality of exploration in the study of attachment in adulthood and infancy. For example, Elliot and Reis (2003) described in their paper: *attachment and exploration* -four studies that support the link between security of attachment and the motivation to explore in an academic context. Feeney and Thrush (2010) found a connection between attachment style and exploration activity in the presence of the spouse, with attachment style being predictive of exploratory behavior. Bernier et al. (2014) reported that secure-autonomous mothers, as measured by the AAI, showed maternal behavior that supports autonomy and exploration when interacting with their child which in turn was related to the child’s secure attachment. The present findings add on to these studies, showing the connection between exploration during a dyadic movement interaction and the attachment of adults.

The MG behavior factor *free* showed significant correlation with the avoidant dimension, but only a trend toward significant correlation with the anxiety dimension. The directions of the correlations were opposite: a high score on the avoidant dimension correlated with a low score on the free factor, and a high score on the anxiety dimension showed a trend toward significance with a high score on the free factor. These results confirm once more that the two dimensions of avoidance and anxiety are different from one another (Mikulincer et al., 2011). In addition, it suggests that the avoidance dimension may be connected to deactivation of exploration. Similar results were reported by Elliot and Reis (2003), who showed that avoidance, rather than anxiety, is negatively linked to exploration.

Looking into the different scales of the MG, the *unusual behavior* scale correlated strongly with insecurity. Eight participants displayed behavior marked as exceptionally bizarre, which could not be captured by any of the other scales and therefore was marked on the scale we named *unusual behavior*. Participants who scored positive on this scale showed odd, out of context, or disoriented behavior in the way they presented themselves and interacted with their partner in the MG. These behaviors resemble that of disorganized infants in the Strange Situation Procedure, who present conflicted and disoriented behavior during reunion with their caregiver (Lyons-Ruth and Jacobvitz, 2016). Previous studies show that the classification of parents as unresolved on the AAI was found to be linked to disorganized attachment classification in infants (Lyons–Ruth et al., 2005). Unresolved classification of parents was also linked to odd, out-of-context behavior of these parents and their adolescents during interaction (Obsuth et al., 2014). Therefore, unusual behavior in the MG may reflect a subcategory of insecurity in our sample. Because of the small sample size, however, in the present study, we grouped together all insecure subgroups, and therefore could not test the nuances of the different insecure categories. A larger sample study is needed to further investigate the meaning of unusual behavior during the MG.

Similar to the results of other studies, the different attachment measures: The ECR dimensions and the AAI classifications did not correlate in our study (Roisman et al., 2007). However, the *free* MG behavior correlated significantly with both the AAI categorical classifications (Main and Goldwyn, 1998, unpublished reference) and the ECR dimensions (Brennan et al., 1998). Each of these measures represents a different school of thought, and the relation between the measurements has been debated both theoretically and empirically (Roisman et al., 2007). The AAI, better represented in developmental psychology, assesses current state of mind regarding childhood experiences (Main et al., 2008). The ECR self-questionnaire, better represented in social psychology, measures the way adults report attachment-related thoughts and feelings regarding adult relationships (Cassidy and Shaver, 2008). In the present study, we showed that the MG behavior correlated with both these measures. These findings suggest that the procedural knowledge manifest in the dyadic movement during the MG expresses both the history of relationships and the way in which relationships are being perceived at present time. Both aspects regarding relationships correlate with the way people move, and therefore are encapsulated in the body.

Our results may imply that the MG behavior could act as a mediator between parental representation (on the AAI) and the quality of parent–child relationship, so that the bodily expression may explain the link in the intergenerational transmission of attachment patterns (Shah et al., 2010). Over 20 years ago, meta-analytic results confirmed the association between caregiver attachment representations and child–caregiver attachment (Van IJzendoorn, 1995). Since then, a large number of studies sought to explore the way in which the mother’s narrative about her childhood history (as measured by the AAI) is transmitted to the child and reflected in the child’s attachment patterns (Verhage et al., 2016), with no final conclusion that fully explains this attachment transmission “gap.” The way parents use their body may carry both their childhood history and their present interaction in relationships with their children, and affect the child’s attachment to the parent. Thus, the parent’s body movement may explain the attachment transmission gap. These assumptions, which need further investigation, have possible clinical implications for designing parent–child interventions that focus on the parent’s body expression.

The significant correlations found in the present study were only with the *free*, not with the *together* factor. The *free* factor explains around 34% of the variance in the MG behavior and therefore seems more substantial than the *together* factor. In future studies, the different scales grouped under the *together* factor can be examined in greater detail separately, to understand how it relates to attachment classification in adulthood. In addition, the correlations between the AAI classification and the *free* factor, although being significant, were underpowered and explained relatively low percentage of the variance therefore needs further investigation in future study with a larger sample.

This preliminary study brings to the fore the practice of drama and dance/movement therapy. Dance/movement and drama therapists base their therapeutic interventions on imitations, and mirroring (Butler, 2012; Ogden and Fisher, 2015; Koehne et al., 2016) using dyadic movement interaction. The present research validates the notion that observing and working on a body level which carry attachment signals can be a meaningful avenue for both assessment and therapeutic processes. More specifically the MG is being used in clinical settings without having been adequately researched. A scoring system can help support further investigation of this technique, both as an assessment tool for progress in intervention and in the design of aims and special areas in movement intervention. This exploratory study is a first step in the development of the MG as a standardized assessment tool. Based on our results, future studies may simplify the coding of the MG and use the classification of *free* as a main dimension, replacing the 19 scales

Our findings showed a connection between the MG behavior and prosocial factors, hence attachment. Therefore, the MG can be used to assess the prosocial abilities of people with specific difficulties in these areas. We recommend examining whether focused practice of the MG improves the performance of the MG behavior and other intervention outcomes, including diminishing of pathological symptoms.

Some of the strengths of the MG, alongside the rich information provided by the implicit (non-verbal) movement expression, are its simplicity and the possibility to bypass the need for verbal report. At the same time, when trying to use the MG as an assessment tool, various motor, and physical limitations, which are not necessarily related to socio-emotional abilities, may act as confounding factors. Therefore, for some people the ability to express their inner world through movement is limited by physical disabilities. Furthermore, movement has a strong cultural component. Our pilot study was conducted in a certain cultural context with a small sample and limited cultural diversity. Although attachment theory received considerable validation in cross-cultural studies (Mesman et al., 2016), further research is needed to validate our findings regarding non-verbal behavior in various cultural contexts.

The present results expand our previous study of one-dimensional movement using the MG device and its relation to attachment classification. Both studies show the connection between dyadic movement and attachment and shed light on exploration as a central characteristic of secure low avoidance adults. Our findings were significant, but showed a small effect size, and thus need further support. Moreover, this pilot study was limited in its distribution of attachment classifications. Having treated the insecure sub-classification as one group, we cannot draw conclusions about the differences between insecure dismissing and insecure preoccupied attachment styles. Follow-up studies with a more representative sample, covering all insecure attachment groups, could help clarify the expression of attachment in the MG more thoroughly, and contribute to the development of the MG as an assessment tool for prosocial abilities in general, and for attachment in particular.

## Author Contributions

RF-S initiated and designed the study. YH helped in developing the MG scales and analyzing the data. NL brought her expertise in dance/movement therapy and assisted in developing the analysis of the movement. NK-K contributed her expertise in attachment research and analyzed all the attachment interviews and conceptualized the results. LN helped to design the study and write the paper.

## Conflict of Interest Statement

The authors declare that the research was conducted in the absence of any commercial or financial relationships that could be construed as a potential conflict of interest. The reviewers EM and KM-C and handling Editor declared their shared affiliation.
