# Supplementary material for: The Body Speaks: Using the Mirror Game to Link Attachment and Non-verbal Behavior
Source: Front Psychol. 2018 Aug 23;9:1560. doi: 10.3389/fpsyg.2018.01560 (PMC6115809; doi:10.3389/fpsyg.2018.01560)
Supplement: Supplementary file 1 [file Table_1.DOCX]

Supplementary material A

Table 1. Descriptive statistics of background variables

1. Categorical Variables

| **%** | **Frequency** |  |  |
| --- | --- | --- | --- |
| 45.8 | 22 | female | Gender |
| 54.2 | 26 | Male |  |
| 56.3 | 28 | Married | Family Status |
| 8.3 | 4 | Divorced |  |
| 18.8 | 9 | In a relation |  |
| 16.7 | 8 | Bachelor |  |
| 91.7 | 44 | Hebrew | Language |
| 8.3 | 4 | Other |  |
| 8.3 | 4 | Orthodox | Belief System |
| 10.4 | 5 | conservative |  |
| 79.2 | 38 | Secular |  |
| 2.1 | 1 | Other |  |
| 29.2 | 14 | Owner | Housing |
| 70.8 | 34 | Rent |  |
| 72.9 | 35 | None | Drama experience |
| 20.8 | 10 | Little |  |
| 6.3 | 3 | Extended |  |
| 66.7 | 32 | None | Dance Experience |
| 29.2 | 14 | Little |  |
| 4.2 | 2 | Extended |  |
| 72.9 | 38 | None | Mirror Game Experience |
| 18.8 | 9 | Little |  |
| 2.1 | 1 | extended |  |
| 75 | 36 | No | Improvisational Experience |
| 20.8 | 10 | Little |  |
| 4.2 | 2 | extended |  |
| 60.4 | 29 | yes | Working out |
| 39.6 | 19 | no |  |

b. Continuous variables

| SD | M |  |
| --- | --- | --- |
| 7.33 | 33.21 | Age |
| 2.56 | 19.54 | Years of Education |
| 1.23 | 1.02 | Number of children |
| 1.35 | 3 | Number of people living at home |
| 1.45 | 3.53 | Number of rooms |

Supplementary material B

**Instructions for the mirror game**

Hello, my name is ___ and thanks for taking part in this study.

We will play together the Mirror Game. This is a game without words. In this game we will create movements together. The aim of the game is simply to produce movement together, like a conversation in movement. This is not a competition. There is no right or wrong. Any movement you make is fine.

In the game there are three rounds, each round is five minutes long. In the first round you will be the “leader” so that you will make movements and I will imitate you accurately. After five minutes we will change roles and I will be the leader and you will imitate me. In the third round we will try to make movements together without designated leader. The ring of the clock will give us a sign to move from to the next round, with no break. Any questions? So we begin.

***Prompting** In case the participant is playing the game wrong or did not understand the rules of the game the experimenter may repeat the instruction one more time.

In case the participant did not understand the game after the second explanation, the experimenter can demonstrate, “leading” for one minute.

In case the participant has more questions during the gam , the experimenter should say: “I will be happy to answer further questions at the end of the game”

Supplementary material C

Table 2: Reliability of the MGC

| **scale** | **ICC** |
| --- | --- |
| Leader/follower | .933*** |
| Breaks | .972*** |
| The ”greeting” | .913*** |
| Fluent/shifts | .911*** |
| Negative affect | .936*** |
| pace | .832*** |
| Eye contact | .891*** |
| Body parts | .895*** |
| Movement directions | .596* |
| Tension/relaxed | .788*** |
| Having fun | .960*** |
| Competitiveness/teasing | .873** |
| Distance | .910** |
| reference to the other | .915*** |
| Shared affect | .956*** |
| Arching | .939*** |
| Gaze aversion | .758** |
| Exploration | .901*** |
| Unusual behavior | .960*** |

p≤.05, *p≤.01, **p≤.005***
